# Development and preliminary user testing of the DCIDA (Dynamic computer interactive decision application) for ‘nudging’ patients towards high quality decisions

**DOI:** 10.1186/1472-6947-14-62

**Published:** 2014-08-01

**Authors:** Nick Bansback, Linda C Li, Larry Lynd, Stirling Bryan

**Affiliations:** 1 School of Population and Public Health, University of British Columbia, 2206 East Mall, Vancouver, BC V6T 1Z3, Canada; 2 Centre for Clinical Epidemiology and Evaluation, Vancouver Coastal Research Institute, 7th Floor, 828 West 10th Avenue, Research Pavilion, Vancouver, BC V5Z 1M9, Canada; 3 Centre for Health Evaluation and Outcome Sciences, St Paul’s Hospital, 588-1081 Burrard St, Vancouver, BC V6Z 1Y6, Canada; 4 Arthritis Research Centre of Canada, 5591 No. 3 Road, Richmond, BC V6X 2C7, Canada; 5 Department of Physiotherapy, University of British Columbia, 212 – 2177 Wesbrook Mall, Vancouver, BC V6T 1Z3, Canada; 6 Faculty of Pharmaceutical Sciences, University of British Columbia, 2405 Wesbrook Mall, Vancouver, BC V6T 1Z3, Canada

**Keywords:** Patient decision aids, Medical decision-making

## Abstract

**Background:**

Patient decision aids (PtDA) are developed to facilitate informed, value-based decisions about health. Research suggests that even when informed with necessary evidence and information, cognitive errors can prevent patients from choosing the option that is most congruent with their own values. We sought to utilize principles of behavioural economics to develop a computer application that presents information from conventional decision aids in a way that reduces these errors, subsequently promoting higher quality decisions.

**Method:**

The Dynamic Computer Interactive Decision Application (DCIDA) was developed to target four common errors that can impede quality decision making with PtDAs: unstable values, order effects, overweighting of rare events, and information overload. Healthy volunteers were recruited to an interview to use three PtDAs converted to the DCIDA on a computer equipped with an eye tracker. Participants were first used a conventional PtDA, and then subsequently used the DCIDA version. User testing was assessed based on whether respondents found the software both usable: evaluated using a) eye-tracking, b) the system usability scale, and c) user verbal responses from a ‘think aloud’ protocol; and useful: evaluated using a) eye-tracking, b) whether preferences for options were changed, and c) and the decisional conflict scale.

**Results:**

Of the 20 participants recruited to the study, 11 were male (55%), the mean age was 35, 18 had at least a high school education (90%), and 8 (40%) had a college or university degree. Eye-tracking results, alongside a mean system usability scale score of 73 (range 68–85), indicated a reasonable degree of usability for the DCIDA. The think aloud study suggested areas for further improvement. The DCIDA also appeared to be useful to participants wherein subjects focused more on the features of the decision that were most important to them (21% increase in time spent focusing on the most important feature). Seven subjects (25%) changed their preferred option when using DCIDA.

**Conclusion:**

Preliminary results suggest that DCIDA has potential to improve the quality of patient decision-making. Next steps include larger studies to test individual components of DCIDA and feasibility testing with patients making real decisions.

## Background

In recent years, numerous patient decision aids (PtDA) have been developed to facilitate informed, value based decisions about treatment options [1]. They have been developed in response to many beneficial treatments or screening strategies which also have negative aspects such as side-effects or high costs. What is best for one patient may be different to another depending on how each values the attributes of each option [2]. Health professionals are often poor proxies of patients’ values [3,4], and often fail appropriately ‘diagnose’ patient preferences [5]. Patients can also have unrealistic expectations of treatment benefits and harms [6].

PtDAs provide facts about the condition, options, and attributes such as outcomes and probabilities for each option; a value clarification task that helps patients evaluate which attributes matter most to them; and a guide in the steps of deliberation and communication required for the informed patient to reach their goal – concordance between what matters most to them and their chosen option [7]. An updated Cochrane systematic review found that, among 115 studies involving 34,444 participants, PtDA increase patients’ knowledge about treatment options, and reduce their decisional conflict related to feeling uninformed and unclear about their personal values [1].

There have been tremendous advances in the way PtDAs are developed, from the way risks are presented [8], to the use of animated stories to better communicate information [9]. However, there has been comparatively little research on reducing decision errors in people using PtDAs [10]. While in theory, PtDAs should help patients identify the best treatment option for them, research shows that various cognitive biases may result in errors that prevent this in some situations [11-14]. For example, individuals are known to make different choices when their options are framed as gains or losses, preferring a surgical procedure with a 90% survival rate to one with a 10% mortality rate [15]. Studies have shown that individual treatment choices are unduly influenced by whether individuals learn first about potential harms or potential benefits [10], and individuals are intimidated and overwhelmed by options that include numerous rare side-effects leading to irrational decisions [16].

The objective of this study was to develop and user test a computer application that enhances conventional PtDAs to improve the quality of decisions by helping patients overcome common decision errors.

### Development of DCIDA

#### Theoretical motivation

Normative decision theory suggests that for patients to approach treatment or screening decisions rationally they need to weigh benefits and harms using deliberative “compensatory strategies” to make trade-offs [17]. That is, a patient can “compensate” for the negative feature of one option by considering a positive feature. A patient looking at cancer screening options may not want to have annual testing, but would not mind if such frequent testing were non-invasive. While this approach helps people identify the treatment option that matches their informed values, descriptive decision theory has identified numerous errors in peoples’ decisions caused by cognitive biases and simplifying heuristics [12-15]. An understanding that people have two systems for cognitive functioning has provided a framework for understanding these errors and providing effective strategies for improving decision making [18]. System 1 refers to people’s intuitive system, which is typically fast, automatic, effortless, implicit, and emotional. While often useful for simple decisions, they can lead to decision errors for more difficult decisions, such as ones requiring compensatory strategies. System 2 refers to reasoning that is slower, conscious, effortful, explicit, and logical. Recent research suggests that when faced with decisions and information that is unfamiliar, complex, or overwhelming – all common traits targeted by most PtDAs – people can switch to use System 1 functioning, which can lead to decision errors [12,13].

By reviewing PtDAs contained in the Ottawa repository [19], the project team identified few examples where PtDAs helped patients making trade-offs, and beyond simple value clarification exercises gave patients little help in choosing what was best for them. The team identified four issues common to nearly all PtDAs that were believed could impede the quality decision-making, and for which interventions were feasible: unstable preferences, order effects, overweighting of rare events, and information overload.

i) *Unstable values:* Unlike most goods and services, where markets help form stable values and consumers understand the relative value of attributes through a process of trial and error and notion of sacrifice, evidence indicates that patients often have unstable values when it comes to health care [20,21]. This follows from the fact that the potential benefits and harms of treatment may be unfamiliar to patients and impossible to evaluate without a great deal of information and reflection. The consequence is that people’s decisions may be inconsistent with their true underlying values.

ii) *Order effects*: For a rational decision maker, the way information is presented should not have an influence on the choice that is made. However, there is evidence that people tend to, for example, remember information presented to them first (primacy effect) or last (recency effect) [22-24]. The decision error in PtDAs caused by this heuristic is demonstrated in a study of women at high risk of breast cancer who were considering tamoxifen, which found that patients who learned first about the risks of the drug thought more favourably of tamoxifen than patients who learned first about the benefits [10]. Nevertheless, harms and benefits must be presented in some order in a PtDA, and therefore, the designer of the PtDA may inadvertently influence the patient to choose a given option.

iii) *Overweighting of rare events*: Most treatments have multiple complications. PtDAs are expected to inform patients about any treatment complication that is reasonably likely to occur. Although there are no absolute criteria regarding which complications must be included for meeting informed consent, most PtDAs show information on any moderately severe complication that occurs at least 1% of the time, and serious complications that occur even less often than that. Prospect theory describes how people systematically overweigh small probabilities in terms of their impact on decisions. The consequence of this phenomenon is that patients using PtDAs can be scared away from treatments with multiple rare side-effects that rationally would have appeared the best treatment option for them [14,16].

iv) *Information overload*: When individuals receive conflicting, incomplete, uncertain, or excessive information, they experience ambiguity and can make contradictory decisions [25]. The role of information overload causing ambiguity in investment decision-making has been well documented. When the complexity of decision-making increases, people tend to expend less effort to actually make their decision, seek others to make decisions for them, or select default options if available [26].

A number of promising strategies have been uncovered for overcoming specific decision errors. One approach is to encourage people to use System 2 thinking instead of System 1 by making the information and decision less overwhelming. This can be achieved by focussing attention on the most pertinent information, and by using analytic processes which reduce the number of ‘internal calculations’ which require cognitive effort [27-30]. In the area of PtDAs, the predominant approach has been to employ formal decision analysis techniques which quantifies patients' values and integrates them with probabilistic information [31-33]. While there are many perceived advantages to this prescriptive approach, there are also criticisms. First, ‘optimal’ options derived from decision analysis are reliant on assumptions, theories and inaccuracies in inputs which mean they may not actually prescribe the best course of action for each patient [34]. Second, the current approaches to decision analysis are typically ‘overt’ to be best course of action, and consequently have been argued to be an extension of paternalism, compromising patient autonomy [35].

An alternative strategy is to leverage System 1 thinking by changing the decision environment to maximize the odds that people will make high quality decisions given known biases [26]. For example, it is known that most people have a bias towards inaction, in which providing a default option has been found to be a powerful decision enhancer [36]. These strategies have increasingly been referred to as ‘nudges’, reflecting the unavoidable paternalistic role of the designer of the tool (in this case, PtDAs) in influencing users’ choices [37]. Nudges have been defined as “…any aspect of the choice architecture that alters people’s behavior in a predictable way without forbidding any options or significantly changing their economic incentives…” [37], and so seek to preserve patient autonomy and freedom of choice.

We sought to employ decision analysis more covertly [38] to improve decision-making by testing various ‘nudges’ which help people focus on the information and options that reflect their values, and simplify their trade-offs.

#### Features

We developed a dynamic computer interactive decision application **(**DCIDA - pronounced ‘decider’) to employ some of the strategies described above. The overarching aim of the DCIDA is to present information and the decision to each person in an individualized way in order to maximize their ability to make choices that reflect their own informed, stable values. Acknowledging that it is rare that any decision support system will induce optimal decisions, the goal is to improve the quality of decisions, from what would be made with conventional PtDAs. Multi-criteria decision analysis (MCDA) using a weighted additive model motivates the application. For treatment decisions, this assumes the preferred option is based on the sum of the importance or *weight* (on a 0–100 scale) of each attribute, say benefit or harm, multiplied by each option’s *score* (on a 0–100 scale) for that attribute. The treatment with the highest weighted score or expected value indicates the patient’s optimal option.

**Figure 1:**
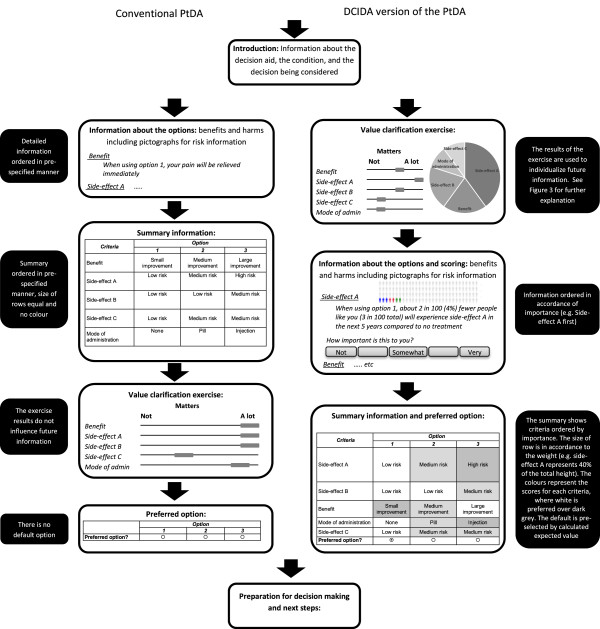
Different pathways for obtaining information and indicating preferences for DCIDA vs conventional PtDA.

**Figure 2:**
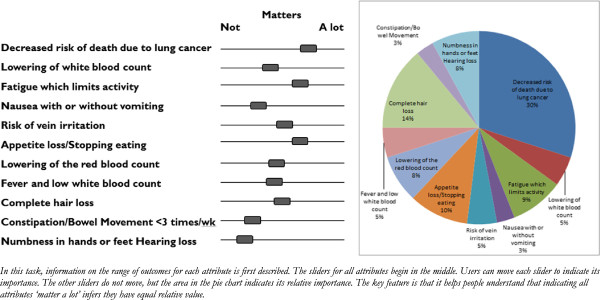
Example of constrained interactive pie chart.

The application contains the same content and information as a PtDA, explaining the condition, providing information about options and their characteristics (benefits, side-effects, costs etc.) using probabilities and pictographs to describe baseline and incremental absolute risks where appropriate, a value clarification exercise, and a summary of information to help the patient deliberate on the decision along with an opportunity to select the preferred option. However, the way this content and information is structured and organized differs, and where possible individualized to make it simpler for each person to choose what is best for them. Figure 1 compares the pathway a patient would take between a conventional and DCIDA version of a PtDA. For example, the first unique feature of the DCIDA is that in *step 1* the value clarification task, which is usually near the end of a conventional PtDA, is moved to the beginning. The objective of the task was to a) provide an opportunity for individuals to reflect on the relative value of attributes and subsequently derive more stable values (see “i) *Unstable values*” above) and b) to generate the *weights* for each attribute for use later in the tool. After preliminary testing, we decided to use an interactive form of constant sum exercise (also known as a “budget pie”) which requires users to allocate a certain number of points (often 100) to each attribute in accordance with the relative importance of each. Constant sum exercises have a long history of use and incorporate a number of properties desirable for encouraging compensatory decisions [39], but have been criticized for requiring a higher levels of numeracy [40]. We developed a simpler version that requires users to move multiple sliders, all linked to an interactive pie chart (Figure 2). For individuals with low numeracy, pie charts can be an effective format for communicating the “gist” of health knowledge and treatment choices [41]. As participants navigated through the following steps of the DCIDA version, they could return at any time to the budget pie exercise to change the weights allocated to different attributes of the decision.

After the values clarification task, *Step 2* presented participants with detailed information (e.g. the actual risk) for each attribute. Where possible, the information was explained in a simple format, including pictographs where necessary to communicate risks. Importantly, this information was ordered in accordance with the rank of the individual’s *weights* – obtained from the value clarification exercise. For each attribute, the patient was asked to rate how important the attribute (outcome such as benefit or harm) for each option. This was used to create the *score* for each attribute. In our preliminary testing we used a simple 5-point scale to derive numerical scores.

**Figure 3:**
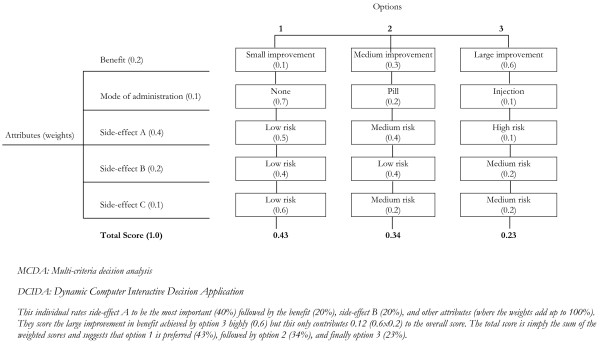
Example of conventional MCDA weights and scores, and total scores and DCIDA.

In *step 3*, the summary information for all consequences was displayed. In contrast to a conventional PtDA: 1) the consequences were ordered in accordance with the rank of the individual’s *weights* – obtained from the value clarification exercise. This aimed to exploit order effects by nudging individuals to focus on the information that would most significantly influence their decision (see “ii) *Order effects”* above); 2) rows were further sized in proportion to the *weights* of each consequence, with the most important consequences being presented in wide rows and less important consequences in narrow rows. This served to take attention away from rare events for the majority of people who rated these attributes to have low importance in the value clarification exercise (see “iii) *Overweighting of rare events”* above); 3) for each consequence, the colour for each option was based on the *score*, with a lighter shade of grey indicating a more preferred option. Colouring aimed to simplify the information presented (akin to traffic light labelling for the nutritional of food [42]), enabling individuals to process multiple pieces of information and distinguish between harms and benefits (see “iv) *Information overload”* above); 4) the sum of the weights and scores were used to determine which option would be preferred using MCDA (Figure 3). This indicated the ‘optimal’ choice for a given individual and became the default option for the participant, helping overcome information overload. On the summary page, users were able to select an option other than the default optimal choice; however the presence of a selected default option has proven to help overcome ambiguity [36].

#### Development

Three PtDAs available to the developers were transformed into DCIDA versions. The goal was to ensure the DCIDA was usable for a broad range of PtDAs, each of which has a different set of individual characteristics. The first PtDA used information on newly developed over the counter medication choices for the treatment of knee osteoarthritis, where it is suspected patients underestimate the risks associated with high doses. The options were no treatment, acetaminophen (lower benefit, but fewer side-effects), and non-steroidal anti-inflammatory drugs (NSAIDs) (greater benefit, but greater side-effects). The second PtDA focussed on treatment options for patients with Obstructive Sleep Apnea (OSA). The primary treatment option is continuous positive airway pressure (CPAP), which is effective but inconvenient to use. Less invasive alternatives such as oral appliances or no treatment are also options. The third PtDA considers chemotherapy options for patients with late stage non-small cell lung cancer (NSCLC). Treatments with higher efficacy are also associated with more frequent and severe side-effects. All three PtDAs were conceived using the Ottawa Framework [43] and were reviewed to International Patient Decision Aid Standards (IPDAS) guidelines [7]. DCIDA versions of each PtDA were made using exactly the same information. An example of the DCIDA version of the PtDAs is available at: http://dcida.cheos.ubc.ca/osa1.

## Methods

### Overview

We focussed our user testing on both usability (whether the user can do what they want to do to without hindrance, hesitation, or questions) and usefulness (does it help the user make a better decision) [44]. Two common approaches to user testing were used – eye-tracking and a think aloud protocol – and these were supplemented with various validated questionnaires. Ethics was granted from University of British Columbia Ethics Board.

### Participants and procedures

A sample of healthy, English-speaking volunteers was recruited through online advertisements and posters. Participants were seated at a computer equipped with an eye-tracker and the interviewer explained the purpose of the study. After gaining consent, the participant went through the calibration procedure to initialize the eye-tracking system. The participant was then asked to choose which clinical scenario they wanted to imagine they were facing: Knee Osteoarthritis, OSA, or NSCLC, and proceed to complete an online version of a conventional PtDA followed by the DCIDA version created for their chosen scenario.

Figure 1 shows the flow for the conventional PtDA. After selecting their preferred option, they were asked to indicate their uncertainty in their decision by completing the Decisional Conflict Scale (DCS). They then went through the DCIDA version (right side of Figure 1) and were again asked to choose their preferred option, and their uncertainty as measured by the DCS. Finally, they were asked to complete the System Usability Scale (SUS) focussing on the DCIDA. After completing the tool, an interview was used to discover users’ impressions about the tool and their experiences. Upon completion of the tasks, the user was compensated with a $25 dollar gift certificate.

### Eye-tracking

Eye tracking is a promising method for usability testing since it can evaluate individuals’ information processing while they deliberate on decisions [45]. It makes it possible to determine what type of information individuals look at and to what extent information is processed. The eye-tracker method is widely used in marketing research as well as research on cognitive processing and decision-making processes [46].

When individuals look at information while reading or searching, they continually make rapid eye movements called saccades. Between saccades, the eyes remain relatively still for approximately 200–500 ms [47]. Individuals do not obtain new information during a saccade because the eyes are moving too quickly. Rather, higher levels of information processing require deeper cognitive processes that can only be processed while the eyes are fixated. Research on information processing is therefore mainly concerned with fixation durations [47]. Fixation durations are assessed by an infrared camera system built into the eye tracker. This camera measures the light reflex on the cornea of individuals sitting in front of the computer screen.

In this study, the eye-tracking data was used for two purposes. First, to assess user experience, we analyzed heatmaps of each page of the tool to ensure respondents were consistently looking and reading the important aspects of design such as instructions. Heatmaps visually display the areas in which fixations on each page occur. Second, to assess usefulness we compared fixations between the conventional display versus the DCIDA display. To analyze the eye-tracker data, we subdivided areas of the summary screen into areas of interest based on each attribute. The time individuals spend looking at relevant information in relation to the total time needed to look at the whole summary information was used to indicate attributes participants were spending time deliberating on [47]. It is expressed by the relative fixation duration, that is, the percentage of the time spent fixating on each attribute relative to the time spent looking at the whole summary information [47]. A Tobii T120 eye tracker embedded in a 17” display was used.

### Decisional conflict scale (DCS)

After stating a preference for one treatment, participants were asked to evaluate their uncertainty in their decision based on a subscale of the DCS [48]. The DCS is a validated scale that assesses patients’ conflict and uncertainty in their decision. While the full scale comprises 16 items divided into 5 subscales – uncertainty, inadequate knowledge, values clarity, lack of support, and ineffective choice – we focussed simply on the uncertainty subscale, the component the DCIDA attempts to increase confidence in the decision. This subscale includes three items: how clear the patient is about the best choice, how sure they feel about that choice, and how easy the choice was to make. All items are reported on a 5 point Likert scale from strongly agree to strongly disagree. Lower scores are desirable as they indicated less conflict. An effect size of 0.06 to 0.3 has been reported to discriminate between decision supporting interventions [49].

### System Usability Scale (SUS)

After completing the tool, participants were asked to answer an adapted version of the SUS. This validated scale asks 10 questions about aspects of user friendliness, content integration, and support needed to answer the tool providing a score between 0 and 100 [50]. The SUS is a commonly used quantitative assessment of usability. It is useful for rough comparison purposes, including assessing the effects of changes from one prototype iteration to the next, and for drawing preliminary conclusions about overall usability of a system. To our knowledge there is no established SUS threshold for usability, however previous studies have shown that a SUS score above 68 is above average for all studies that have used the scale, while a score above 74 would place the system in the top 30% [51]. We chose to adopt these thresholds for our study.

### Think aloud study

We used a verbal protocol analysis, a form of ‘think aloud’ technique, to further investigate respondents’ choices [52]. Think aloud data can be obtained in two ways: *concurrent*, where respondents are asked to verbalize their thoughts as they complete a task, and *retrospective*, where respondents are asked to describe what they were thinking after the task was completed. Following experience from previous studies, we used a hybrid approach whereby respondents were asked to think aloud as they completed the tool, however if they did not think aloud for a period of 10 seconds, the interviewer would ask them to reflect back on their choices [53]. This approach interferes less with respondents’ thought processes while still allowing an exploration of how respondents were making choices. Respondents were asked not to explain or plan what they were saying, but to act as if they were speaking to themselves. Following the survey, the interviewer asked respondents debriefing questions. In general, respondents were asked how they found the information and choices they were presented with and how they would improve the tool. The interviews were tape-recorded and later transcribed. Responses were coded by each step in the tool and whether they were related to user experience or usability. Two independent reviewers then coded the valence of each comment (e.g. positive, negative or neutral) and differences resolved by discussion.

## Results

### Participants

In total, 20 participants were recruited via posters and completed the study. Eleven participants were male (55%), a 15 participants were white, and the mean age was 35 (range 19–59) (Table 1). All but two participants had at least a high school education and 8 (40%) had a college or university degree. None of the participants were suffering from a serious illness and only three participants were currently taking prescription medications. Twelve respondents chose to complete the OSA version and four each chose the NSCLC and Osteoarthritis versions.

**Table 1:** Participant characteristics

	**Value**
Age, mean (range)	35.2 (19-59)
Sex, % male	55%
Race, n%	
White	15 (75%)
Asian	5 (25%)
Education, n %	
At least high school	18 (90%)
At least college or university graduate	8 (40%)

### User experience

#### Eye-tracking

In general, heat maps suggested participants were reading all the relevant information on each page. For the first 4 participants, it was noted that there were few fixations on the titles of the scales of the value clarification task (whether each attribute was more or less important). The titles were increased in size and bolded, and this led to increased fixations in subsequent participants.

#### SUS

The mean SUS score for DCIDA was 73 and ranged from 68 to 85. This suggests that all participants considered the tool better than average interfaces and that the tool has reasonable usability overall. The lowest scores related to a perception that the tool was unnecessarily complex. There was no difference between the different PtDA versions.

#### Think aloud analysis

In total, the think aloud analysis yielded 65 comments relating to user-experience. Positive comments were generally around the interactive features of the tool and its ease of moving from step to step. The subject of negative comments included the amount of words required to read, the wording of key instructions and a lack of intuitiveness in how to interact with some features (such as the value clarification exercise). Overall, 16 out of 20 (80%) stated they had no major issues while using the tool. The 4 participants that suggested the tool was difficult to use were all in the oldest age quartile. Points of improvement included: provision of examples to show how to interact with key features (9 out of the 20 [45%]), clearer colours, speed of the software, and wording of certain questions (all less than 25% of participants).

### Usefulness

#### Eye-tracking

**Figure 4:**
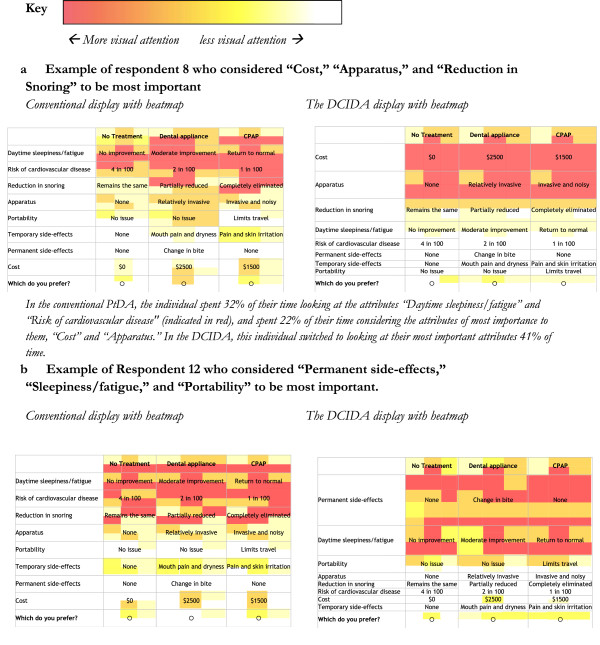
Heatmaps of 2 examplar respondents (where colour represents the proportion of time spent fixating in areas within the defined cell space).

Regardless of the type of summary, we observed an order effect whereby respondents spent more time observing the attributes at the top of the list (23% of time spent on first attribute) versus bottom of the list (13% of the time spent on last attribute). This influenced the amount of time individuals fixated on attributes they felt were more important to them (Table 2). In the conventional summary, 18% of fixation duration was spent on the most important attribute, followed by 16% of duration on the second most important attribute. The DCIDA summary demonstrated an increase to 30% and 18% respectively. Similarly, in the conventional display, 12% of time was spent on the least important attribute, compared to only 5% of time using the DCIDA. Analyzing the subgroup of participants that changed their preferred option between the conventional and DCIDA summaries shows even greater differences in fixations (Table 2). The heatmaps in Figure 4 describe the influence of DCIDA on two individuals.

**Table 2:** Results of system usability scale, decisional conflict, and eye tracking

	**Conventional**	**DCIDA**
**System Usability Score, mean (range)**	-	74 (68–85)
**Decisional conflict uncertainty subscale (0 = good, 4 = bad),**		
I am clear about the best choice for me	2.9	2.3
I feel sure about what to choose	2.8	2.2
This decision is easy for me to make	3.4	3.1
Overall score	50.4	38.3
**Mean fixation duration (secs)**		
Most important attribute	4.7	5.6
2^nd^ most important attribute	4.2	3.3
Least important attribute	3.1	1.1
Other attributes	14.1	8.5
Other areas of the screen	16.2	12.8
Total	42.3	31.3
**Mean relative fixation**		
Most important attribute	18%	30%
2^nd^ most important attribute	16%	18%
Least important attribute	12%	6%
**Relative fixation – in 7 participants changed preference**		
Most important attribute	17%	34%
2^nd^ most important attribute	14%	19%
Least important attribute	10%	5%

#### Quantitative responses

For the 12 participants using the OSA tool, based on the conventional display, 5 participants chose the Oral Appliance while 6 chose CPAP. Two respondents were ‘very sure’ of their decision, while 4 respondents were ‘not very sure,’ with the rest being ‘moderately’ or ‘somewhat sure.’ When presented with the DCIDA version of the summary information, 4 of the 12 participants changed their preferred option. The results for the cancer and osteoarthritis tools produced similar results with 2 participants changing their decision for the cancer tool, 1 for the osteoarthritis tool. Overall, the decisional conflict uncertainty subscore was 50.4 for the conventional summary, reducing to 38.3 in the DCIDA version (Table 2).

#### Think aloud analysis

Of the 45 comments coded for usefulness, 28 (62%) were positive. The predominant positive themes were that the treatment information was easy to access and the DCIDA summary information either confirmed or improved their treatment choice. Negative comments were from individuals who felt that they knew their decision and were frustrated that they had to negotiate all the steps before indicating their preference option. Four of the participants suggested that they felt ‘nudged’ to one of the choices, in a positive frame. When prompted for further clarity, it was not clear whether they felt this had encroached their autonomy to choose or not.

## Discussion

This explorative study investigated the feasibility of using nudges based on MCDA to improve the quality of treatment decisions. Software was developed to enable information from conventional PtDAs to be restructured in how it is presented to the user through dynamic and interactive interfaces. The software was tested for both usability and usefulness in 20 participants. Both aspects of user testing provided some positive results, and provide important information for future development.

There is limited research on the use of behavioural economic approaches to improve patients’ use of PtDA. A study by Ubel et al. found that order effects in decision aids could be unbiased by providing the patient further information (in graphical form) [10]. This is one approach for encouraging System 2 thinking, yet a concern with targeting numerous biases through this general technique is that providing more information can sometimes overwhelm people, causing them to revert back to System 1 thinking [54]. The DCIDA approach has sought to enable users to read less information, but focus on information that will most likely influence their choice.

While there has been substantial attention to ‘nudge’ theory in health [55,56], to our knowledge this theory has not been tested in PtDAs. Default options have become the predominant ‘nudge’ used in health interventions to date [57], but have typically selected a single default option for *all* users. For example, organ donation programs may ‘nudge’ patients to enrol by making organ donation the default option. It has been proposed that nudges could be used in PtDAs for conditions where the evidence clearly indicates that one treatment option is superior to the others [58]. This is controversial as most PtDAs are developed for preference sensitive decisions where two or more medically appropriate options exist, and they seek to promote rather than diminish patient autonomy. DCIDA has been designed as a bridge between non prescriptive PtDAs and overtly prescriptive decision analysis tools.

The objective of this study was to examine if there was a difference in response between the two versions of PtDAs. If no difference was observed, we would reject the hypothesis that the DCIDA version had any impact. While we establish some preliminary demonstration of effectiveness, this study alone cannot ascertain whether the impact is real or useful and should be interpreted with caution for three primary reasons. First, participants considered the conventional summary before the DCIDA version, therefore an ordering effect might have been observed whereby they became more informed as they spent more time viewing the information. On average 42 seconds was spent viewing the conventional display versus 31 seconds on the DCIDA display. It is also difficult to disentangle the effects of each aspect of the DCIDA version that differed from the conventional summary, such as the values clarification exercise, the layout, or colour hues. While it would be unfeasible to investigate the impact of each design feature, we have subsequently evaluated the impact of ordering effects in a larger controlled study [59]. A second limitation relates to putting the values clarification exercise at the beginning of the decision aid. We deliberately presented this exercise up front to elicit more stable values from participants. However, recent studies have suggested that it can be problematic to engage in importance weighting too soon in the decision-making process [60,61]. A related third reason is that we do not know if participants who changed their decision actually made an improved choice. This challenge of measuring the quality of patients choices is a limitation in all research on PtDAs [62].

Further, we acknowledge some limitations to our use of standard measures. With regard to the System Usability Scale (SUS), this measure has been used frequently for usability evaluations of Internet-based interfaces; it is not used typically for evaluations of Internet-based PtDAs. Validation of the scale was based on studies of interfaces up to 18 years old and, thus, there are contextual and design differences between DCIDA and the average tools used to validate the SUS. However, by triangulating the results of our SUS scores with our think aloud and eye-tracking results, we determined that the initial prototype has acceptable usability, though we aim to improve it in future iterations of the prototype and to use and report a newer iteration of the SUS developed by Bangor et al. [63]. Additionally, we chose to use the Uncertainty subscale of the Decisional Conflict Scale and acknowledge that our analysis would have benefitted from also using the Values Clarity subscale [49]. Use of this additional measure would have contributed to our understanding of how DCIDA impacted participants’ ability to arrive at stable values.

While the usability scores and improvements in decisional conflict are encouraging, they suggest there is still opportunity to further improve the tool. At the time data collection, the DCIDA software was in alpha stage, and the results of this research have motivated us to move to a different platform for the beta version. The higher usability results may also be due to the hypothetical nature of the task. Participants did not have the diseases and were aware they were testing a tool. In addition, subjects were majority college-educated who had access to and comfort using computers. These findings may not be generalizable to a more heterogeneous population with lower education or computer proficiency. We are also cognisant that it will be important for the software to be compatible with Internet use on tablets, which will require separate testing.

Given these opportunities for future research, we plan to further explore the influence of the DCIDA in subsequent studies. In these, individuals will be randomized between a conventional PtDA and a DCIDA version and plan to determine if DCIDA’s unique design features lead to improvements in decision quality, including concordance between what matters most to individual patients and their chosen option. We propose to consider carefully how value concordance is measured. There are no standard criteria for studying values concordance and a recent Cochrane review [1] shows that there is substantial heterogeneity among the measures that authors have used to date. We agree with the growing number of researchers calling for further study into the “active ingredients” of values clarification [64] and the creation of standard measures for analyzing values congruence [62,65]. Such research will assist us in identifying what proportion of people make values congruent decisions when they use DCIDA in comparison to conventional tools. We also plan to ask questions about patients’ attitudes to the role of nudges in making autonomous decisions [38]. Finally, we believe it will be crucial to include patients of varying health literacy, and numeracy to examine the influence of the tool in different patient groups.

## Conclusion

The DCIDA has been developed to enhance conventional PtDAs to assist patients in choosing the treatment that is most congruent with their informed values. This paper reports on the theoretical motivation for the DCIDA and then describes an experiment in which the tool is user tested. The results give some empirical support that the DCIDA is understandable to users and that it can help users focus on attributes that are of individual importance to them – to the extent that some participants changed their decisions. A number of valuable insights were learned for improving the next version of the DCIDA. In conclusion, we propose that the DCIDA is a promising approach to improve conventional PtDAs. Further development is required to improve its usability and usefulness; however research on testing preliminary effectiveness on patient decision-making is justified.

## Abbreviations

PtDA: Patient decision aids; DCIDA: Dynamic computer interactive decision application; NSCLC: Non-small cell lung cancer; IPDAS: International patient decision aid standards; SUS: System usability scale; DCS: Decisional conflict scale; OSA: Obstructive sleep apnea; CPAP: Continuous positive airway pressure; NSAIDs: Non-steroidal anti-inflammatory drugs.

## Competing interests

Dr Bansback was funded by Pfizer Canada for postdoctoral research relating to the development of methods for improving decisions. Funding was unrestricted and had no bearing on the treatments considered.

## Authors’ contributions

NB conceived the project, carried out the interviews, performed statistical analysis, and drafted the manuscript. LCL participated in the study design and help drafted the manuscript. LL and SB helped conceive the project, interpret the statistical analysis, and help draft the manuscript. All authors read and approved the final manuscript.

## Pre-publication history

The pre-publication history for this paper can be accessed here:

http://www.biomedcentral.com/1472-6947/14/62/prepub
